# Cost and Effects of Different Admission Screening Strategies to Control the Spread of Methicillin-resistant *Staphylococcus aureus*

**DOI:** 10.1371/journal.pcbi.1002874

**Published:** 2013-02-21

**Authors:** Tanya Gurieva, Martin C. J. Bootsma, Marc J. M. Bonten

**Affiliations:** 1 Julius Center for Health Research & Primary Care, University Medical Center Utrecht, Utrecht, The Netherlands; 2 Faculty of Science, Department of Mathematics, Utrecht University, Utrecht, The Netherlands; 3 Department of Medical Microbiology, University Medical Center Utrecht, Utrecht, The Netherlands; Imperial College London, United Kingdom

## Abstract

Nosocomial infection rates due to antibiotic-resistant bacteriae, e.g., methicillin-resistant *Staphylococcus aureus* (MRSA) remain high in most countries. Screening for MRSA carriage followed by barrier precautions for documented carriers (so-called screen and isolate (S&I)) has been successful in some, but not all settings. Moreover, different strategies have been proposed, but comparative studies determining their relative effects and costs are not available. We, therefore, used a mathematical model to evaluate the effect and costs of different S&I strategies and to identify the critical parameters for this outcome. The dynamic stochastic simulation model consists of 3 hospitals with general wards and intensive care units (ICUs) and incorporates readmission of carriers of MRSA. Patient flow between ICUs and wards was based on real observations. Baseline prevalence of MRSA was set at 20% in ICUs and hospital-wide at 5%; ranges of costs and infection rates were based on published data. Four S&I strategies were compared to a do-nothing scenario: S&I of previously documented carriers (“flagged” patients); S&I of flagged patients and ICU admissions; S&I of flagged and group of “frequent” patients; S&I of all hospital admissions (universal screening). Evaluated levels of efficacy of S&I were 10%, 25%, 50% and 100%. Our model predicts that S&I of flagged and S&I of flagged and ICU patients are the most cost-saving strategies with fastest return of investment. For low isolation efficacy universal screening and S&I of flagged and “frequent” patients may never become cost-saving. Universal screening is predicted to prevent hardly more infections than S&I of flagged and “frequent” patients, albeit at higher costs. Whether an intervention becomes cost-saving within 10 years critically depends on costs per infection in ICU, costs of screening and isolation efficacy.

## Author Summary

Within hospitals antibiotic-resistance of bacteria is common and it complicates treatment of bacterial infections. Screening of patients on admission for carriage of methicillin-resistant *Staphylococcus aureus* (MRSA) allows for strategies where carriers are treated with barrier precautions, e.g., isolation in single-bedrooms. At least theoretically, this should prevent spread of these bacteria. Several screen-and-isolate studies have been performed. However, the outcome was not unequivocal, possibly because clinical trials to determine the optimal screening strategy would necessitate long periods of follow-up due to stochasticity. In the absence of direct evidence we have used mathematical modelling to quantify the theoretical effectiveness and expenses of different screen-and-isolate strategies in hospitals with a high prevalence of antibiotic-resistant bacteria. We find that a strategy to screen patients who were previously known as carriers, possibly combined with screening of ICU-patients is the most cost-saving strategy for the best estimate of isolation efficacy of 25%. With a high efficacy of isolation all strategies are expected to become cost-saving compared to the do-nothing scenario.

## Introduction

Methicillin-resistant *Staphylococcus aureus (MRSA)* may cause severe infections in hospitalized patients, such as bloodstream infections, surgical wound infections and pneumonia. These infections are associated with increased mortality rates, longer length of hospital stay and higher health care costs compared to methicillin-sensitive strains [1]. Typically, such infections are most prevalent in intensive care units (ICUs) [2]. Patient to patient transmission via – temporarily – contaminated hands of health care workers is considered an important mode of spread [3]. Therefore, prevention of nosocomial spread has been focused on reducing transmission opportunities through isolation measures and enhanced adherence to basic infection control practices, such as hand hygiene [4]. Nevertheless, despite multiple guidelines recommending these practices, infection rates due to MRSA remain high in most countries [5], [6].

It has become increasingly clear that rapid identification of carriage of MRSA, followed by implementation of barrier precautions for carriers, could be a powerful tool in controlling nosocomial spread [7]–[10]. However, screening all patients admitted to the hospital (universal screening) imposes a huge (financial) burden on a hospital system, and its benefits have not been unequivocally demonstrated [11]–[13]. Other screening strategies may, therefore, be more cost-beneficial, such as screening of ICU admissions only, screening of certain high-risk patients or screening of patients who were detected as MRSA-carriers at previous admissions. The optimal screening strategy may differ between settings, but evidence for the most cost-effective strategy in each setting is lacking. As a result, screening strategies vary substantially between hospitals, even within countries. Experimental trials to determine the optimal screening strategy for each of those settings would necessitate long periods of follow-up and huge financial investments. For such complex problems in the absence of direct evidence, mathematical modelling might offer the best alternative to quantify theoretical effectiveness and expenses of different screening strategies in different settings [14].

Here we have performed multiple scenario analyses of a mathematical model to compare the effects and costs of different “screen and isolate” (S&I) strategies, with special emphasis of such a strategy in ICU populations.

## Methods

### Simulation model

We have used an extended version of a previously described dynamic stochastic simulation model that contains three hospitals of 693 beds, each with an extramural population of 220,000 subjects [9]. Upon hospitalization, patients are usually admitted to “their own” hospital, but sometimes to one of the other hospitals (ratio 38 to 1). Each hospital comprises two types of wards: 36 18-bed normal wards with five health care workers (HCWs) per ward and five 9-bed Intensive Care Units (ICUs) with nine HCWs per ICU and 80 HCW per hospital with non-restricted patient contacts. After 8-hours, each shift of HCWs is replaced and HCWs are confined to a single ward during each shift. Upon hospitalization patients can be admitted to both types of wards. One of the most important changes of the model [9] is a change in the length of stay and mortality of patients in the different wards. In ICUs, 70% of the patients stay, on average, 1.5 days, with an ICU mortality of 2% per stay. After ICU discharge, these patients stay, on average, seven days in non-ICU wards, before hospital discharge. The remaining 30% of ICU-patients stay, on average, 10 days in ICU and have an ICU-mortality of 25% per stay. The ICU survivors remain hospitalized for, on average, 15 days in non-ICU wards. Patients without ICU admission stay on average 7 days. These parameters are based on patient data from a multi-center ICU study in the Netherlands [15]. Apart from transfer from ICUs to other wards, patients can be transferred between non-ICU wards, from non-ICU wards to ICUs, between ICUs, and between hospitals, all with different rates. Most important model parameters are shown in Table 1.

**Table 1:** Model parameters.

Parameter	Default value	Source
Average length of stay* in ICU (70% of admissions) (days)	1.5	[15]
Average length of stay* in ICU (30% of admissions) (days)	10	[15]
Average length of stay* in general wards after ICU-discharge for ICU-survivors (70%) (days)	7	[15]
Average length of stay* in general wards after ICU-discharge for ICU-survivors (30%) (days)	15	[15]
Average length* of stay for patients without ICU admission (days)	7	UMC**
ICU-mortality of short stay ICU-admissions (70% of admissions)	2%	[15]
ICU-mortality of long stay admissions to ICU (30% of admissions)	25%	[15]
Non-ICU mortality	2%	[15]
Staff : patient ratio in ICU	1∶1	UMC**
Staff : patient ratio in non-ICU ward	5∶18	UMC**
Staff : patient ratio of HCWs not restricted to a ward	01∶08.70	UMC**
Duration of colonization in extramural population (days)	370	[40], [41]
Transmission risk ICU: transmission risk in non-ICU wards	3∶1	Assumption
Specificity of rapid diagnostic test	96%	[21], [22]
Sensitivity of rapid diagnostic test	93%	[21], [22]
Turnaround time of conventional microbiological test	1 day	[21], [22]
Specificity of conventional microbiological test	100%	Gold standard
Sensitivity of conventional microbiological test	100%	Gold standard
Turnaround time of conventional microbiological test	4 days	[21], [22]
Daily MRSA detection rate by clinical cultures in non-ICU wards	0.03	UMC**
Daily MRSA detection rate by clinical cultures in ICU	0.3	UMC**
Cost of RDT+ conventional test at admission (range)	20€ (2–102)	[31]
Incremental costs of an isolation day (range)	20€ (2–102)	[31]
Costs of an infection in an ICU (range)	30k€ (1–40)	[30]
Costs of an infection in a non-ICU ward (range)	1k€ (0.5–2.5)	[30]
Daily infection risk for a colonized patients in ICU (range)	0.7% (0.14%–1.4%)	[27], [28]
Compliance of admission screening	88%	UMC**

* The length of stay is geometrically distributed.

** UMC parameters are estimated from data from the University Medical Center Utrecht, the Netherlands.

Individuals are also subdivided into “frequent” patients and “occasional” patients, distinguished by hospitalization rates of once per year (frequent) and once per ten years (occasional) (average sizes in the population being 20,000 belonging to the “frequent” group and 200,000 to the “occasional” group). Patients from either group can be admitted to both non-ICU and ICU wards and the mortality rate during hospitalization is the same for both groups. As a result, on average, 50% of the hospital population consists of “frequent” patients. In this study we use the “frequent” group as a high-risk population for MRSA carriage, and one of the possible screening strategies includes screening of “frequent” patients. All patients are either carrier of MRSA or uncolonized and susceptible for colonization and 1% of the colonized patients is 10 times more infectious (so-called superspreaders).

Infection control interventions are not based on the true colonization status, but on the available documentation of the colonization status only. Patients either (1) have documented carriage, (2) are not suspected of MRSA colonization (but could still be colonized), or (3) are suspected of colonization, e.g., after documented carriage during previous hospitalization or because of risk factors for MRSA carriage. Throughout this paper the latter patient category will be labelled as “flagged” patients. Importantly, we assume that the pathogen predominantly spreads in hospitals through cross-transmission and that there is hardly any spread in the community. Transmission occurs primarily between patients and HCW in the same ward, but occurs also, at a much lower rate, between wards. Transmission parameters are chosen such that the per admission reproduction number *R_A_* [16] is around 1.1 and 0.3 for ICU and non-ICU wards respectively, which corresponds in our do-nothing scenario to an endemic prevalence of 5% hospital-wide and of 20% in ICUs. Although most estimates of the prevalence of MRSA in ICUs and hospital wards are slightly lower than our values [17], in some ICUs MRSA-prevalence of 20% is not uncommon even with isolation measures [18].

MRSA blood stream infections may impact LOS, as was shown, by de Kraker et al. [1] and Wolkewitz et al. [19]. However, the attributable mortality and LOS due to MRSA colonization is limited [20]. As most patients colonized with MRSA do not have overt infections, the transmission dynamics of MRSA will be dominated by these patients. We, therefore, have chosen not to explicitly incorporate the additional LOS in patients with overt infections in the model, but to incorporate these additional LOS in the costs associated with an MRSA infection.

Results are based on 1,000 independent runs of the stochastic simulation model for a period of 10 years after implementation of interventions.

### Screening for carriage

The microbiological screening method is, in all simulations, a rapid diagnostic test with turnaround time of 1 day and sensitivity and specificity of 93% and 96%, respectively [21], [22]. This is supplemented with conventional microbiological cultures with a turnaround time of 4 days and an assumed sensitivity and specificity of 100%. The conventional culture results are used as backup to correct false-negative and false-positive results of the rapid diagnostic test. MRSA carriers that are not detected by screening (due to absence of screening, false-negative results or acquisition of MRSA after screening) can be identified as carrier when conventional microbiological cultures, i.e., with a turnaround time of 4 days, are performed for clinical reasons at a rate of 0.03 and 0.3 per patient day for non-ICU wards and ICUs, respectively. The main reason for taking clinical cultures is the presence of fever.

We consider four different S&I strategies that are compared to a do-nothing scenario without any active screening at admission. In all four S&I strategies patients identified as MRSA-carrier will be “flagged” as such. The flagged status will be removed when such a patient has a negative conventional culture. The following S&I strategies are considered:

1. S&I of flagged patients at hospital admission
2. S&I of all patients at ICU admission and flagged patients at hospital admission
3. S&I of “frequent” patients and flagged patients at hospital admission
4. S&I of all patients at hospital admission (universal screening).

In all scenarios we assume that 12% of the admission screenings that should be performed according to the strategy are missed. In each scenario patients documented as carrier will be treated in isolation, which reduces the likelihood of transmission by 100% (perfect isolation), 50%, 25% or 10%, with 25% as default value [18], [23]. Screening of flagged patients, e.g., patients with a history of MRSA colonization [24], [25] and screening of ICU patients both are strategies that are used in hospitals across the world [12], [18].

Screening of “frequent” patients is not a strategy that is currently applied. Yet, since previous hospitalization is associated with MRSA colonization [26], we have chosen this strategy as an intermediate between screening flagged patients only and universal screening.

Although no limits to isolation capacity are assumed, we keep track of the number of patients in isolation to determine the volume of isolation capacity needed. The daily probability to develop an infection for a colonized patient is set at 0.7% and 0.2% in ICU and non-ICU wards, respectively, with sensitivity analysis ranges of (0.14%–1.4%) and (0.1%–0.3%) respectively. This implies that on average 3% and 1.4% of all patients in ICU and non-ICU wards will develop an infection in the do-nothing scenario (Table 1) [27], [28].

### Estimates of expenses

We estimated the costs of the different S&I strategies for a hospital using a 3% inflation rate per year. The analysis was performed from a hospital perspective and costs are reported in Euros using the price level of 2010. The default incremental costs from a hospital perspective of these infections (including a costs of prolonged due to MRSA-infection length of stay) were €30,000 in ICU and €1,000 in non-ICU wards, with ranges for sensitivity analysis of (€1,000–€40,000) and (€500–€2,500) respectively [29], [30]. The costs of a screening test performed at admission ranged from €2 to €102 with €20 as default value [31]. The incremental costs of treating a patient one day in isolation varied from €2 to €102 with €20 as default value [31].

For every S&I strategy and set of costs we determined (see supplementary Text S1) the time till the mean daily costs with the intervention strategy became lower than the mean daily costs in the do-nothing scenario (denoted as *T*). The 90% credibility intervals denote the uncertainty due to the inherent stochasticity of the dynamics of MRSA (with 5% of simulations yielding higher and 5% yielding lower results than the credibility interval).

### Sensitivity analyses

Univariate sensitivity analyses were performed for all costs, the discount rate, and the probability to develop an infection. For the parameters with the highest sensitivity on results in the univariate analysis we investigated the dependence of *T* on the parameters.

## Results

**Figure 1:**
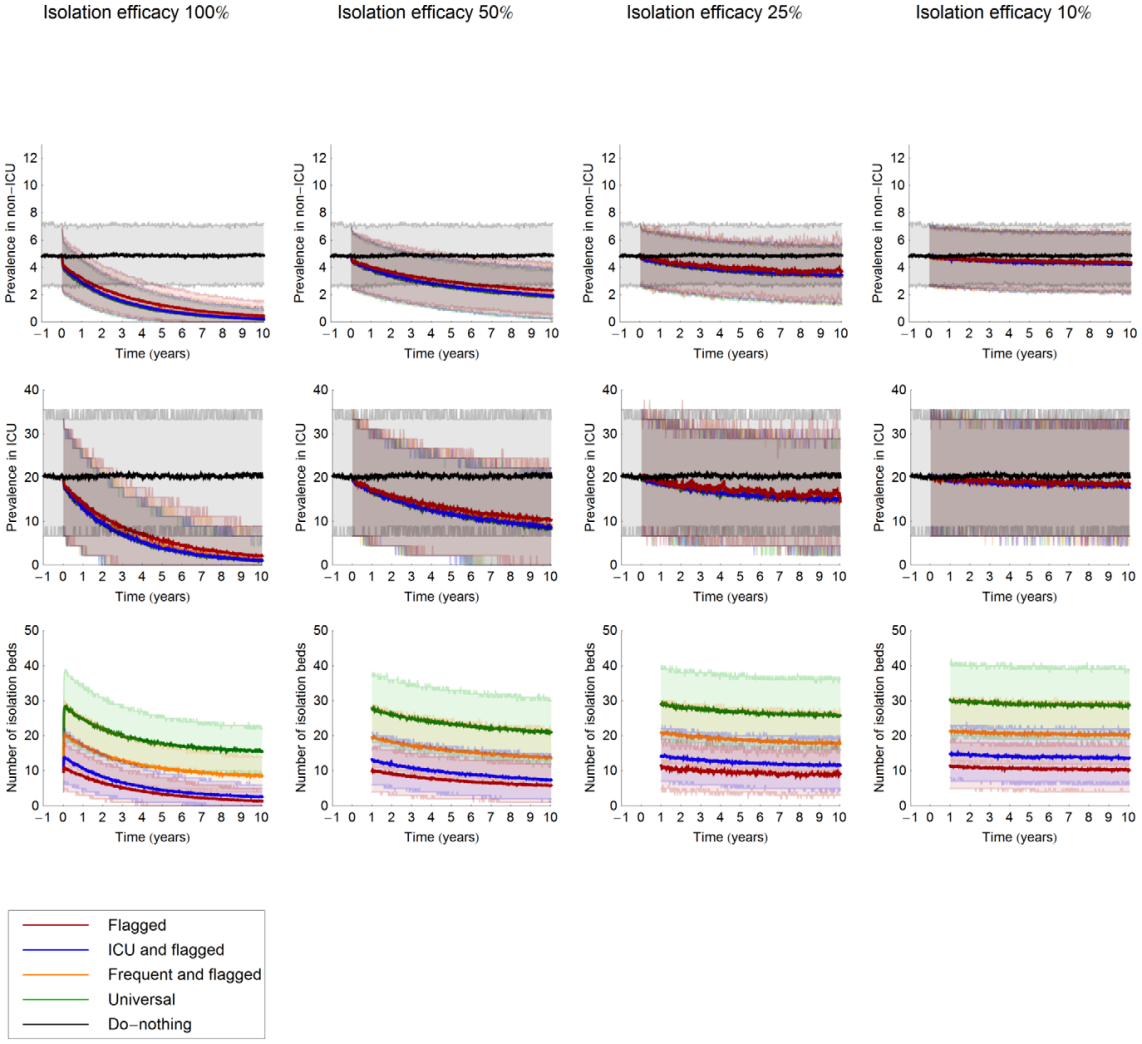
Prevalence of MRSA hospital-wide, in ICU wards and the number of isolation beds needed. The upper graphs denote the hospital-wide MRSA prevalence for different values of the isolation efficacy. The middle row of graphs depicts the prevalence of MRSA in ICU wards. The lower row of graphs depicts the number of isolation beds needed hospital-wide. Interventions start at time 0 and the lines for negative time correspond to the “do-nothing” scenario. Efficacy of patient isolation varied from left to right from 100%, 50%, 25% to 10%. The lines denote the mean of 1000 simulations; the coloured shaded areas denote the 90% credibility intervals due to stochasticity. All parameter values are at the default-value.

The model predicts a decrease of the mean hospital-wide prevalence of MRSA in five years after the start of the interventions from 5% to, depending on the strategy, a value between 3.7–3.9% when isolation efficacy is 25% and 0.8–1.2%, 2.5–2.9% and 4.3–4.5% when isolation efficacy is 100%, 50% and 10%, respectively (Figure 1). The mean prevalence in ICU is predicted to decrease from 20% to 15.9–17.2% for isolation efficacy of 25% and 3.8–5.6%, 11.6–13.0% and 18.4–19.2% for isolation efficacy 100%, 50% and 10%, respectively. Ten years after the start of the intervention, the hospital-wide prevalence is predicted to be 0.2–0.5%, 1.9–2.3%, 3.3–3.8% and 4.2–4.4% and the mean prevalence in ICU predicted to be 1.0–2.2%, 8.5–10.5%, 14.7–16.5% and 18.2–18.5% for an isolation efficacy 100%, 50%, 25% and 10%, respectively (Table 2). Naturally, universal screening leads to the largest decline in the prevalence, while S&I of flagged patients results in the smallest decline.

**Table 2:** Results of the interventions for the default parameter values.

	Type of the intervention (targeted screening + isolation)				
Efficacy of isolation (%)	No intervention	Flagged only	ICU + flagged	“frequent” + flagged	Universal hospital
Mean intervention costs (tests + isolation costs) during 10 years (in millions €)					
100	0	0.179	0.845	3.27	6.29
50		0.23	0.909	3.32	6.35
25		0.26	0.949	3.35	6.38
10		0.279	0.972	3.37	6.4
Mean total Costs (intervention costs + costs of infections) during 10 years (in millions €)					
100	7.3	2.7	2.9	5.4	8.3
50		5.1	5.3	7.9	10.7
25		6.5	6.9	9.3	12.3
10		7.1	7.7	10	13.2
Mean prevalence in ICU 10 years after start of the intervention (%)					
100	20	2.2	1.1	1.3	1
50		10.5	8.6	9.2	8.5
25		16.5	15.0	15	14.7
10		18.5	18.2	18.2	18.2
Mean number of infections prevented in ICU over 10 years					
100	0	152	168	163	170
50		76	89	86	91
25		32	40	39	43
10		11	14	14	14
Mean number of infections prevented in non-ICU wards over 10 years					
100	0	131	142	141	146
50		76	86	86	90
25		41	48	48	51
10		22	25	25	25
Mean costs of intervention per infection prevented (in kilo €)					
100	-	0.6	2.7	10.8	19.9
50		1.5	5.2	19.3	35.1
25		3.6	10.8	38.5	67.9
10		8.4	24.9	86.3	164
Mean savings as compared to the do-nothing scenario during 10 years (in millions €)					
100	0	4.6	4.4	1.9	−0.96
50		2.2	1.96	−0.56	−3.4
25		0.85	0.42	−2.0	−4.9
10		0.19	−0.4	−2.8	−5.9
Median time till the daily expenses become less than in the do-nothing scenario (*T*) (years)					
100	-	<0.1	<0.1	1.6	5.4
50		<0.1	0.7	7.4	>10
25		3.3	6.0	>10	>10
10		7.6	>10	>10	>10
Mean screening and isolation costs (in millions €) till *T* (max 10 years)					
100	-	<0.1	<0.1	0.53	3.4
50		<0.1	0.1	2.5	>6.35
25		0.1	0.6	>3.35	>6.38
10		0.2	>0.97	>3.37	>6.4

Only when isolation efficacy >50% the strategy to screen flagged and ICU patients is predicted to reduce the prevalence in both ICU and non-ICU units more than screening flagged and “frequent” patients (Table 2). Universal screening leads to slightly lower prevalence in 10 years than other strategies. However, when isolation efficacy is low (10%), universal S&I is hardly more effective. S&I flagged patients only is less effective for all considered values of isolation efficacy. Differences in effects of interventions will increase with higher initial prevalence of MRSA (data are not shown).

Naturally, the number of isolation days needed varies considerably with the strategies and the isolation efficacy. The number of isolation beds needed increases immediately after the start of the intervention, most prominently for universal screening (Figure 1). The peak of the mean number of isolation days required for universal screening is 2.5, 2 and 1.4 times higher, as compared to screening flagged patients only, screening of flagged and ICU patients and screening of flagged and “frequent” patients, almost independently of isolation efficacy.

**Figure 2:**
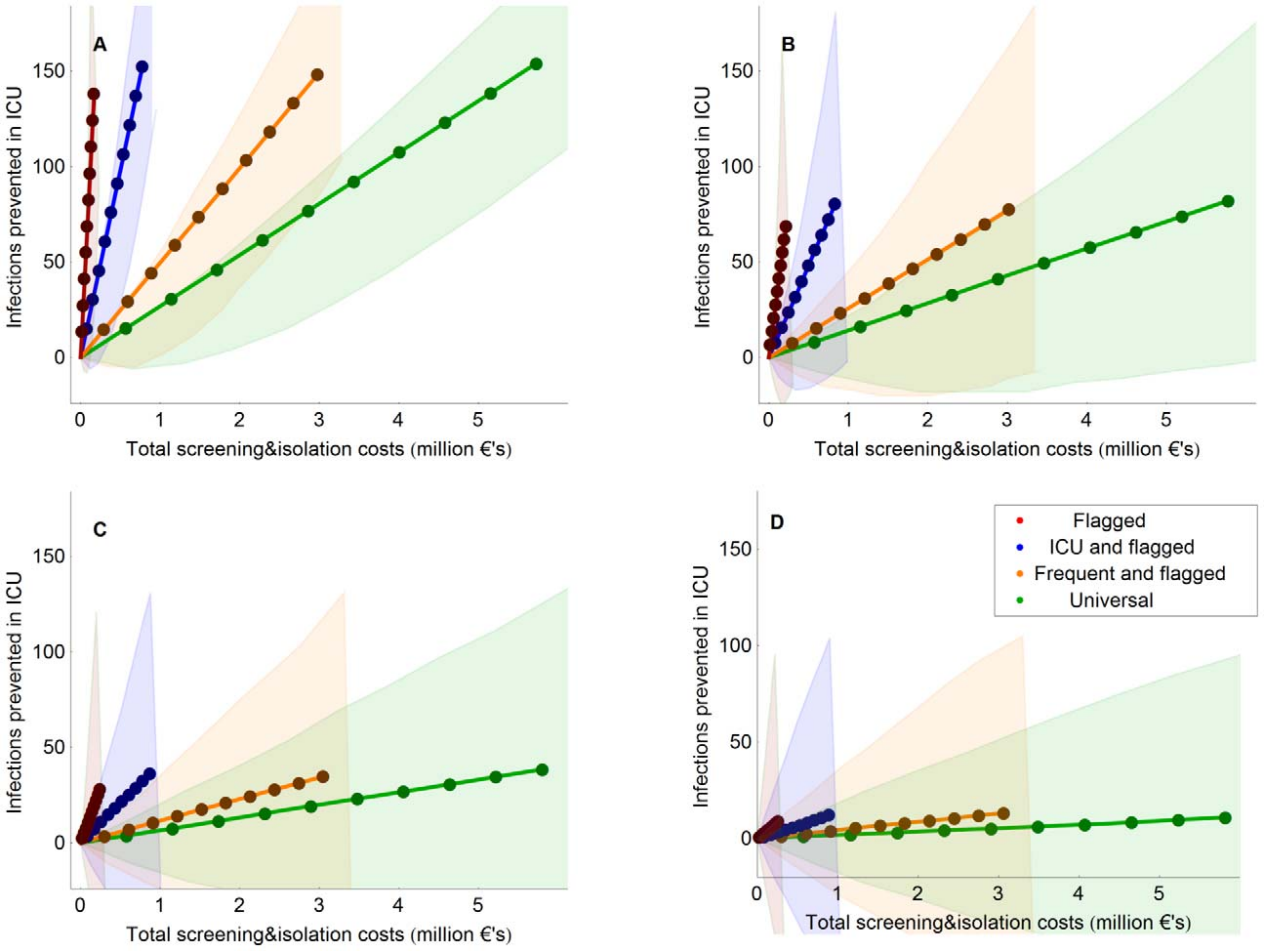
Number of infections prevented in ICUs and the cost of the intervention during the first 10 years after implementation. Isolation efficacy was 100% (A), 50% (B), 25% (C) and 10% (D). The credibility intervals denote the uncertainty due to the inherent stochasticity of the dynamics of MRSA and contain 90% of our simulation results. The 10 dots correspond to the means after 1,2,…,10 years.

With isolation efficacy of 100%, 50%, 25% and 10%, screening of flagged and ICU patients will prevent on average 310, 175, 88 and 39 infections per hospital (168, 89, 40 and 14 in ICU) in 10 years time at the costs of €845.000, €909.000, €949.000 and €972.000 (Table 2). The costs of intervention measures per infection averted were lowest for screening of flagged patients only, being €632, €1.529, €3.598 and €8.447 for isolation efficacy levels of 100%, 50%, 25% and 10%, respectively. Universal screening was associated with the highest costs per infection averted, i.e., €19.918, €35.056, €67.857 and €164.093 for isolation efficacy levels of 100%, 50%, 25% and 10%, respectively. We have also compared the predicted number of infections prevented in ICUs and hospital, and the financial consequences of different strategies in high-endemicity settings (Figure 2 and Figure S1 in supplementary).

Whether a strategy will become cost-saving from the hospital perspective, as compared to the do-nothing scenario, critically depends on the isolation efficacy and the costs per infection averted. With our default efficacy of isolation of 25%, only two strategies are expected to be cost-saving within 10 years: screening of flagged and ICU patients and screening of flagged patients only. The expected total gain in 10 years time is estimated to be €420.000 and €850.000 respectively (Table 2). When efficacy of isolation is 10%, screening of flagged patients only is the only cost-saving strategy within a time window of 10 years. Universal screening is not expected to be cost-saving within 10 years even if efficacy of isolation is 100%.

**Figure 3:**
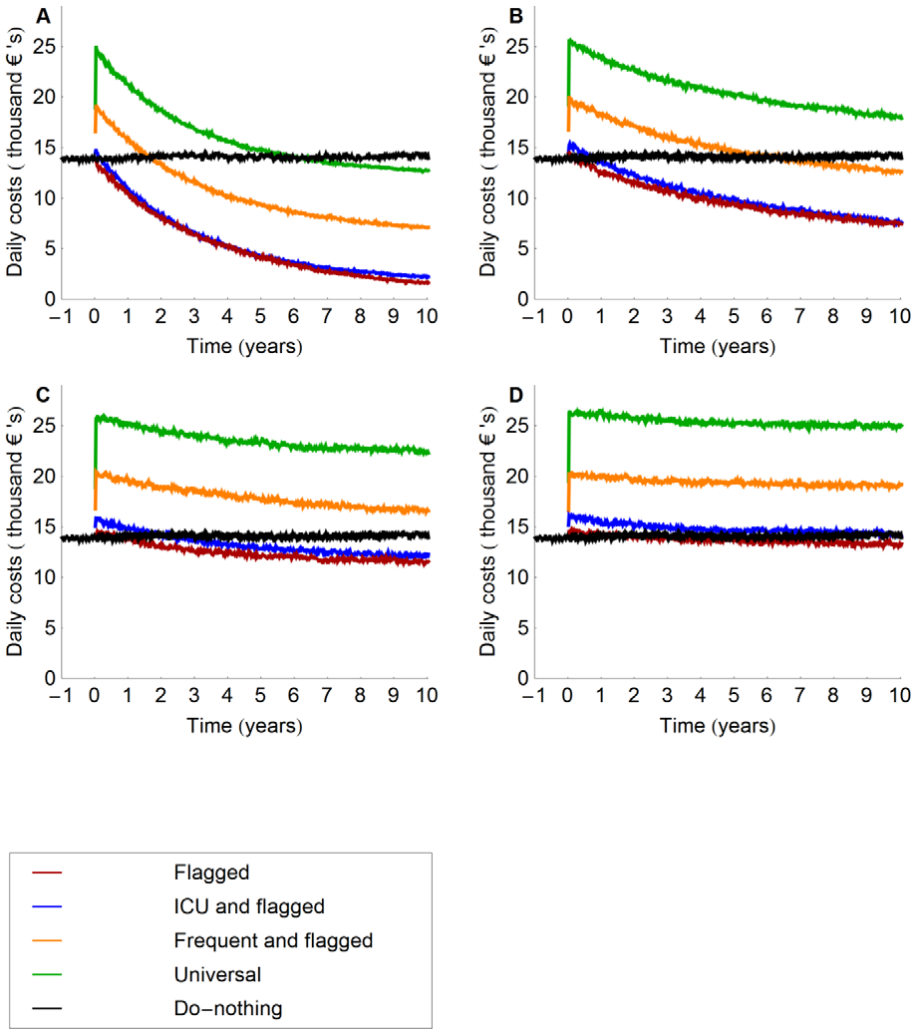
Mean total daily costs (intervention costs and costs due to infections) for different intervention strategies. Isolation efficacy was 100% (A), 50% (B), 25% (C) and 10% (D) and all other parameter values are at the default value (see Table 1). Credibility intervals are not shown because of large fluctuations in the daily costs due to stochasticity.

In the do-nothing scenario the number of infections caused by MRSA, and, therefore, the costs associated with these infections, will be –more or less– constant in time (Figure 3). The costs associated with the intervention will initially lead to increased hospital costs. Yet, due to prevention of infections the hospital costs per unit of time will decrease and may – at a time *T* – become lower than in the do-nothing scenario (Figure 3); For all values of the efficacy of isolation, our model indicates that *T* is minimal for screening of flagged and ICU patients and screening of flagged patients only. Universal screening has the largest value of *T*, which is only below 10 years when isolation efficacy is 100% (Figure 3 and Table 2).

### Sensitivity analyses

**Figure 4:**
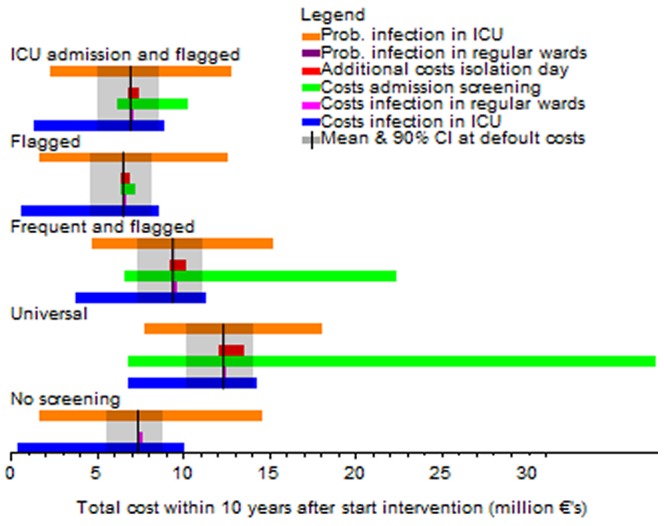
Univariate sensitivity analysis of the total costs during the first 10 years after implementation of the intervention when the isolation efficacy is 25%. The black line corresponds to the mean costs for the default parameter (see Table 1) and the grey area corresponds to the 90% credibility interval at the default values. All coloured bars correspond to the range of the mean total costs of an intervention strategy if one parameter is changed between its extreme ranges (Table 2).

Univariate sensitivity analyses indicate that the total costs are rather insensitive to the costs of isolation, the costs per MRSA infection in non-ICU wards and the probability to develop an infection in non-ICU wards (Figure 4, supplementary figures S2, S3, S4). However, the total costs are sensitive to the costs associated with an infection in ICU wards and the probability per day for a colonized patient to develop infection in ICU wards. The dependence of total costs on the costs per screening test varies between screening strategies and is highest for universal screening.

**Figure 5:**
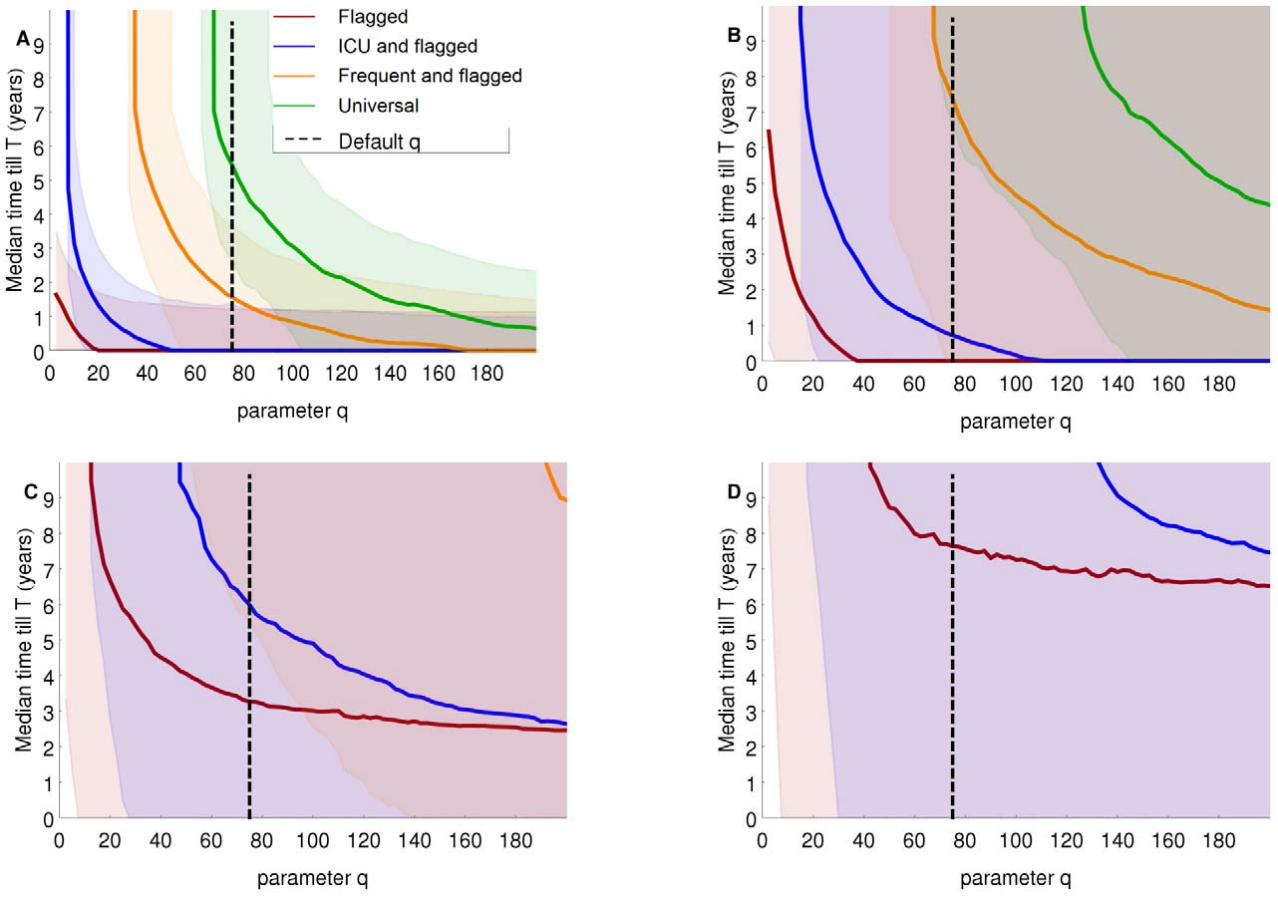
Time (*T*) till the median (and 10% and 90% quantile) weekly total costs with different intervention scenarios become lower than in the do-nothing scenario. The parameter *q* on the horizontal axis is the infection costs per colonized patient day in ICU wards divided by the costs of a single screening at admission. Isolation efficacy is A) 100%, B) 50%, C) 25% and D) 10%. The costs of an infection in non-ICU wards was set at €1.000 and additional costs of an isolation day at €20. If a curve for a strategy is not depicted in the figure, the median time till the weekly costs of the strategy become lower than the weekly costs in the do-nothing scenario exceeds 10 years.

Naturally the number of infections and the costs due to infections are more or less proportional to the number of colonized patient days. We, therefore, define a “constant of proportionality” as the “cost of an infection in ICU wards multiplied by the probability per day to develop an infection in ICU wards”. This constant can be interpreted as the costs due to infections per colonized patient day in an ICU ward. The total costs of the interventions are sensitive to “the costs due to an infection per colonized patient day in an ICU ward” divided by the costs of a single screening test performed at admission (Figure 5). We denote the ratio of these two costs by *q*. When screening is cheap and the costs of infections in ICU are high (*q* is large), all four strategies will have lower daily costs as compared to the do-nothing scenario within 10 years (*T*<10 years) for high values of isolation efficacy. With isolation efficacies of 25% or 10% only S&I of flagged and S&I of flagged and ICU patients will reach *T* within 10 years in the considered range of *q*. With decreasing values of *q*, the time *T* increases and at some critical value of *q*, the number of infections prevented by a strategy becomes too low to compensate for the costs of the intervention. This critical value of *q* depends on the strategy and the isolation efficacy (Figure 5).

The value of *T* is relatively insensitive to the other costs. Only for relatively low infection costs per colonized patient day in ICU and high costs per test, the costs per isolation day will significantly impact *T* (data not shown).

Changing the discount rate to either 2% or 4% hardly influenced the results.

## Discussion

Using a dynamic stochastic simulation model, we have evaluated four intervention scenarios to control the spread of MRSA under comparable *in silico* conditions. Although universal screening at hospital admission leads to the fastest decline in both the hospital-wide and ICU prevalence of MRSA, it also requires the highest investment costs and the longest time till return of investment. In our analyses, screening all patients at ICU admission and those previously detected with MRSA (so-called flagged patients) or screening of flagged patients only were almost equally cost-saving in a 10 years period and were both associated with the fastest return of investment. These strategies should, therefore, be seriously considered by hospitals that aim to control the nosocomial spread of MRSA.

Our findings are complimentary to those of two other modelling studies on screening for carriage with antibiotic-resistant bacteria in hospitalized patients. In one study, Hubben and co-workers compared the effects of PCR-based and chromogenic screening tests [32]. Determination of the optimal screening was not investigated in the current study, and we have, therefore, used a fixed time-to-result parameter. In the other modelling study, Robotham and co-workers investigated the effects of different screening tests in ICU patients, in combination with patient isolation and decolonisation [33]. The latter study did not include the effects of ICU-screening on the non-ICU hospital population and did not include the possibility of patients being readmitted while still colonized.

Yet, this is an important aspect of the dynamics of nosocomial MRSA as it explains why control measures may have not only a direct, almost instantaneous, effect on the prevalence of the nosocomial MRSA in the hospital, but also an indirect effect due to interruption of the so-called feedback loop; when less patients acquire colonization during hospitalization, less patients will be colonized upon readmission to the hospital (see supplementary Figure S5). This lower admission prevalence in time ensures that controlling spread of the nosocomial MRSA will become easier in time. Therefore, neglecting these feedback loop dynamics will underestimate the cost-savingness of interventions.

An important assumption of our model is that the pathogen spreads predominantly in health care settings. Interventions in health care settings will not be very effective in prevention of acquisitions in the community. With substantial spread in the community, a smaller fraction of the acquisitions can be prevented and also the fraction of the patients colonized on admission that are flagged will reduce. In the extreme case that transmission almost exclusively occurs outside health care settings, interventions in hospitals are ineffective and the cheapest strategy is the optimal one. For these reasons, our model is not applicable for community-associated MRSA, but is applicable for other pathogens with similar epidemiological characteristics as MRSA.

Although reductions in the occurrence of nosocomial MRSA infections have been reported [7], [10], multi-resistant Gram-negative bacteria, such as those producing extended-spectrum β-lactamases (ESBL) or carbapenemases are emerging in health care settings worldwide [34]. With no new antibiotics on the horizon to treat infections caused by these bacteria, effective transmission control strategies are needed. Yet, identifying the most effective control strategy for every possible setting through clinical trials seems impossible. Well-designed large clinical trials on rapid diagnostic testing of MRSA yielded highly variable results, varying from no effects on infection rates in surgical units [11], [12], [35] to 69.6% reductions in hospital-wide infection rates [10]. Moreover, the stochastic nature of ARB dynamics necessitates long study periods to avoid that conclusions are primarily based on chance events, rather than on true effects. We have, therefore, used mathematical modelling. Of note, mathematical models always are a simplification of real life complexities and cannot produce very precise predictions for a certain situation. For instance, we have assumed that all isolation measures were equally effective in all isolated patients and that all measures were executed with equal efficacy. One can easily think of scenarios in which these assumptions do not hold [36]. Therefore, the main value of modelling is the comparison of different scenario analyses, while keeping other important parameters constant, rather than providing exact values.

In doing so, our analyses identified screening of flagged patients and ICU patients as a very powerful control strategy, even reducing prevalence levels in non-ICU wards. The central role of the ICU in our model follows from two assumptions. First, many patients discharged from ICU are transferred to other wards. Therefore, prevention of spread in ICUs will reduce the frequency at which MRSA is introduced in other wards. Second, the likelihood of cross-transmission is higher in ICUs than in non-ICU wards. This assumption is motivated by the more frequent (and possibly even more intense) contacts between patients and HCWs, allowing HCWs to act as transmission vectors of MRSA. Moreover, antibiotic selective pressure is higher in ICUs than in non-ICU wards, which may increase the likelihood that a HCW will pick up a pathogen during a physical patient contact and that another patient will be successfully colonized after being contacted by a temporarily contaminated HCW. Finally, the severity of disease of critically ill patients in ICU wards makes them more susceptible to acquire colonization with MRSA than patients in non-ICU wards. Several studies indeed support the potential effects of ICU-screening on hospital-wide resistance levels [37].

With regard to the costs of interventions, our analyses were most sensitive to the costs associated with an ICU-acquired infection caused by MRSA. Many studies have quantified the costs of ICU-acquired bacteremia and ventilator-associated pneumonia [30] and these estimates were all in the range of the €30,000 that we used. However, these costs sensitively depend on the additional length of stay that can be ascribed to infections, which is difficult to determine, see e.g. [38], [39]. Another important aspect is the role of the ICU in the patient flow. We have used data on patient admissions to 13 ICUs in the Netherlands. Naturally, patient flow may be different in other hospitals.

One of the simplification of the model is that patients should be colonized with MRSA before they are at risk of getting an infection with MRSA, i.e., we did not explicitly incorporate that some patients may acquire MRSA infection directly without being colonized first, i.e., due to invasive medical procedures. A slight increase in the daily probability for colonized patients to acquire an infection would lead to the same ratio of colonized and infected patients. Therefore, our sensitivity analysis on the daily probability for colonized patients to acquire an infection can also be interpreted as a proxy for a sensitivity analysis to the parameter which determines how often patients acquire an infection without being colonized.

We also assumed that the rates of conventional microbiological cultures performed for clinical reasons are independent of screening on admission (0.03 and 0.3 per patient day in non-ICU and ICU wards). We have assumed that a clinical suspicion of infection is the main reason for obtaining clinical cultures, and that screening for MRSA-carriage on admission reduces the frequency of obtaining clinical cultures in case of a clinical suspicion of infection.

Our model contains many parameters and some parameter values are unknown, whereas others may differ between hospitals and countries. We have based our values on data from the literature and from our own hospital, where possible. To fully capture the effects of parameter uncertainty we would have considered to perform a probabilistic sensitivity analysis (PSA) for all parameters simultaneously, as was performed by Robotham et al. [33]. However, due to the higher complexity of our simulation model, as compared to the model of Robotham et al., this was computationally unfeasible. We, therefore, had to restrict our sensitivity analysis primarily to univariate sensitivity analysis. As a result, there may be more uncertainty in the results as we have presented here.

We did not include decolonization of detected carriers as a measure to control MRSA. Naturally, adding this measure (if successful at low costs) would increase intervention effects and would make the duration till return of investments shorter. Although persistently colonized HCWs were included as potential sources for MRSA transmission, we did not include screening and decolonization of them as intervention measure. This intervention measure would - in most settings – only slightly enhance the control of MRSA transmission, at the cost of significant expenses due to the necessity to replace colonized HCWs.

The (cost)-efficacy of admission screening strategies critically depends on the effectiveness of the infection prevention measures taken when a carrier of MRSA is detected. If these measures are not very effective, it may not be wise to invest lots of efforts in detecting carriers. The effectiveness of barrier precautions has been sufficiently high in the Netherlands and the Scandinavian countries to prevent high prevalence levels of MRSA. However, it is still debated whether patient isolation prevents transmission at all [23], and a recent estimate indicated that the efficacy is in the order of 25% [18], We, therefore, advocate to perform more clinical studies to determine the efficacy of decolonization, isolation or cohorting measures in different settings.

In conclusion, our study demonstrates marked and robust differences in the costs and effects of different infection control measures for MRSA. Because of the central role of ICU wards in patient flow in hospitals, the vulnerability of ICU patients to infections caused by MRSA and the high costs associated with these infections targeted infection control measures in ICU wards are likely to be the most effective and cost-saving from a hospital perspective.

## Supporting Information

Figure S1 **Number of infections prevented in ICUs and the cost of the intervention during the first 5 years after implementation in a high-endemicity settings (14% hospital-wide prevalence).** Isolation efficacy was 100% (A), 50% (B), 25% (C) and 10% (D). The credibility intervals denote the uncertainty due to the inherent stochasticity of the dynamics of MRSA and contain 90% of our simulation results. The dots correspond to the means after 1,2,.. years.
(TIF)

Figure S2 **Univariate sensitivity analysis of the total costs during the first 10 years after implementation of the intervention when the isolation efficacy is 100%.** The black line corresponds to the mean costs for the default parameter (see Table 1) and the grey area corresponds to the 90% credibility interval at the default values. All coloured bars correspond to the range of the mean total costs of an intervention strategy if one parameter is changed between its extreme ranges (Table 2).
(TIF)

Figure S3 **Univariate sensitivity analysis of the total costs during the first 10 years after implementation of the intervention when the isolation efficacy is 50%.** The black line corresponds to the mean costs for the default parameter (see Table 1) and the grey area corresponds to the 90% credibility interval at the default values due. All coloured bars correspond to the range of the mean total costs of an intervention strategy if one parameter is changed between its extreme ranges (Table 2).
(TIF)

Figure S4 **Univariate sensitivity analysis of the total costs during the first 10 years after implementation of the intervention when the isolation efficacy is 10%.** The black line corresponds to the mean costs for the default parameter (see Table 1) and the grey area corresponds to the 90% credibility interval at the default values. All coloured bars correspond to the range of the mean total costs of an intervention strategy if one parameter is changed between its extreme ranges (Table 2).
(TIF)

Figure S5 **Admission prevalence of MRSA and the annual attributable mortality rates as function of the time since start of the intervention.** The upper graphs denote the dynamics of the MRSA prevalence at admission for different values of the isolation efficacy . The lower graphs depict the dynamics of the annual attributable mortality rates. Both the admission prevalence and the attributable mortality e decrease due to the so-called feedback loop. Interventions in hospital start at time 0 and the lines for negative time correspond to the “do-nothing” scenario. Efficacy of patient isolation varies from left to right from 100%, 50%, 25% to 10%. The lines denote the mean of 1000 simulations; the coloured shaded areas denote the 90% credibility intervals due to stochasticity. All parameter values are at the default-value.
(TIF)

Text S1 Explanation of the calculation of the time T. Abstract in Dutch. Abstract in Russian.
(DOC)
