# Supplementary figures and images for: Cost and Effects of Different Admission Screening Strategies to Control the Spread of Methicillin-resistant Staphylococcus aureus
Source: PLoS Comput Biol. 2013 Feb 21;9(2):e1002874. doi: 10.1371/journal.pcbi.1002874 (PMC3578746; doi:10.1371/journal.pcbi.1002874)

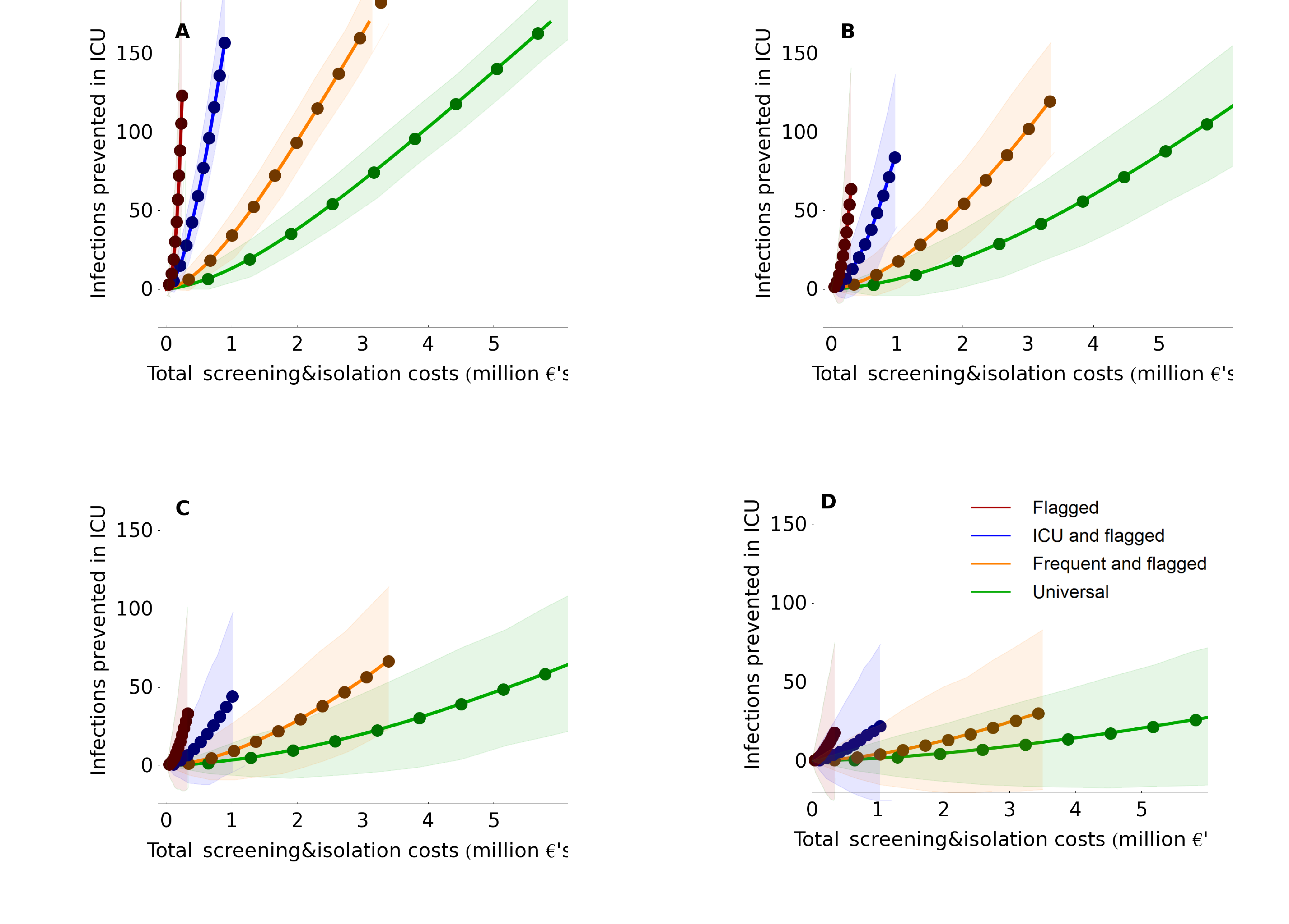

Supplement: Figure S1 — Number of infections prevented in ICUs and the cost of the intervention during the first 5 years after implementation in a high-endemicity settings (14% hospital-wide prevalence). Isolation efficacy was 100% (A), 50% (B), 25% (C) and 10% (D). The credibility intervals denote the uncertainty due to the inherent stochasticity of the dynamics of MRSA and contain 90% of our simulation results. The dots correspond to the means after 1,2,.. years. (TIF) [file pcbi.1002874.s001.tif]

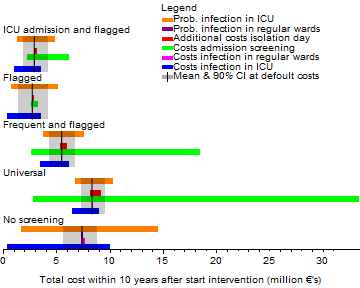

Supplement: Figure S2 — Univariate sensitivity analysis of the total costs during the first 10 years after implementation of the intervention when the isolation efficacy is 100%. The black line corresponds to the mean costs for the default parameter (see Table 1) and the grey area corresponds to the 90% credibility interval at the default values. All coloured bars correspond to the range of the mean total costs of an intervention strategy if one parameter is changed between its extreme ranges (Table 2). (TIF) [file pcbi.1002874.s002.tif]

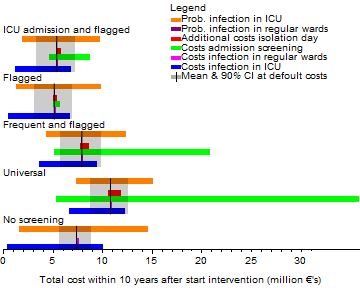

Supplement: Figure S3 — Univariate sensitivity analysis of the total costs during the first 10 years after implementation of the intervention when the isolation efficacy is 50%. The black line corresponds to the mean costs for the default parameter (see Table 1) and the grey area corresponds to the 90% credibility interval at the default values due. All coloured bars correspond to the range of the mean total costs of an intervention strategy if one parameter is changed between its extreme ranges (Table 2). (TIF) [file pcbi.1002874.s003.tif]

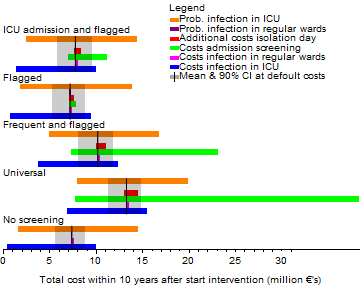

Supplement: Figure S4 — Univariate sensitivity analysis of the total costs during the first 10 years after implementation of the intervention when the isolation efficacy is 10%. The black line corresponds to the mean costs for the default parameter (see Table 1) and the grey area corresponds to the 90% credibility interval at the default values. All coloured bars correspond to the range of the mean total costs of an intervention strategy if one parameter is changed between its extreme ranges (Table 2). (TIF) [file pcbi.1002874.s004.tif]

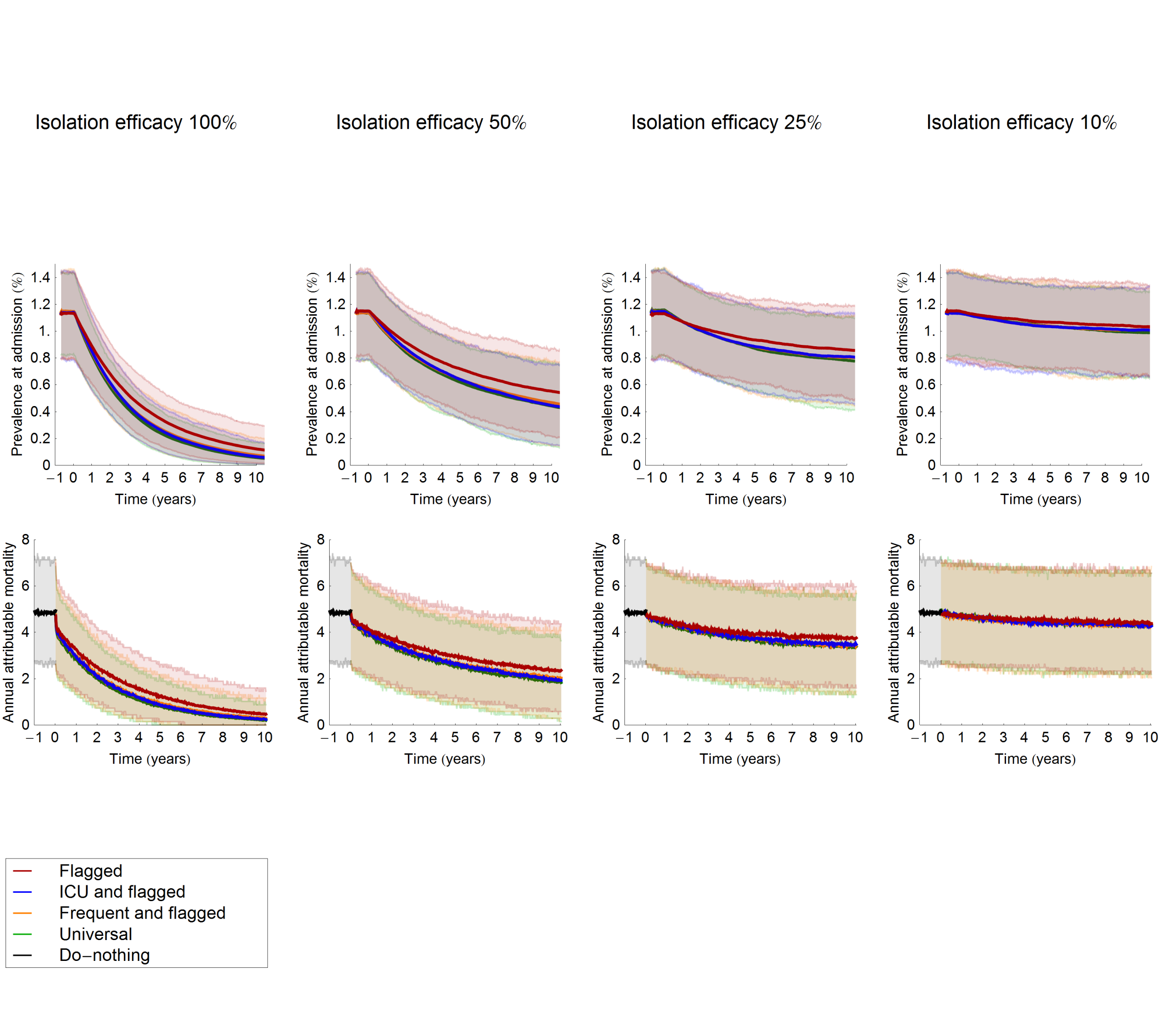

Supplement: Figure S5 — Admission prevalence of MRSA and the annual attributable mortality rates as function of the time since start of the intervention. The upper graphs denote the dynamics of the MRSA prevalence at admission for different values of the isolation efficacy . The lower graphs depict the dynamics of the annual attributable mortality rates. Both the admission prevalence and the attributable mortality e decrease due to the so-called feedback loop. Interventions in hospital start at time 0 and the lines for negative time correspond to the “do-nothing” scenario. Efficacy of patient isolation varies from left to right from 100%, 50%, 25% to 10%. The lines denote the mean of 1000 simulations; the coloured shaded areas denote the 90% credibility intervals due to stochasticity. All parameter values are at the default-value. (TIF) [file pcbi.1002874.s005.tif]
