# Supplementary material for: Cost and Effects of Different Admission Screening Strategies to Control the Spread of Methicillin-resistant Staphylococcus aureus
Source: PLoS Comput Biol. 2013 Feb 21;9(2):e1002874. doi: 10.1371/journal.pcbi.1002874 (PMC3578746; doi:10.1371/journal.pcbi.1002874)
Supplement: Text S1 — Explanation of the calculation of the time T. Abstract in Dutch. Abstract in Russian. (DOC) [file pcbi.1002874.s006.doc]

# Supplementary Text S1

by

T. Gurieva, M.C.J. Bootsma, M.J.M. Bonten

**Explanation of the calculation of the time *T***

For each strategy we define the time *T* as the median time of 1000 simulations till the daily total costs (of intervention and associated with infections) becomes lower than the mean daily costs in the do-nothing scenario. In the latter scenario the mean total costs are constant in time, but depend on the ratio *q* of the “costs due to an infection per colonized patient day in ICU divided by cost of a single screening”.

Due to chance events, both the number of tests performed and the number of infections per week fluctuate per simulation, and so do the weekly total costs. To determine the time *T* for a single simulation, we performed a linear regression analysis for the weekly costs in a certain time window. The left point of the time window is the latest week where all weekly costs before that week exceed the mean weekly costs in the “do-nothing” scenario. The right point of the time window is defined by the first week for which all weekly costs after that week are lower than the mean weekly costs in the “do-nothing” scenario. This leads to 1000 values of *T,* some of which may exceed 10 years. In Figure 5 we plotted median values (and 10% and 90% quantiles) of time *T* as function of the ratio *q*.

**Abstract in Dutch (Samenvatting in het Nederlands).**

Ziekenhuisinfecties met bacteriën resistent voor antibiotica, bijvoorbeeld meticilline-resistente *Staphylococcus aureus* (MRSA) komen veel voor in de meeste landen. Het zogenaamde screening en isolatie (S&I)) beleid waarbij screening voor MRSA-dragerschap wordt gecombineerd met het in isolatie verplegen van bekende dragers van MRSA is niet in alle omstandigheden succesvol gebleken. Verscheidene S&I strategieën zijn gesuggereerd, maar er zijn geen klinische studies die de kosten en het effect van deze strategieën vergelijken. Daarom hebben we een wiskundig model gebruikt om te bepalen welke parameters de meeste invloed hebben op de kosten en effecten van verschillende S&I strategieën

Het stochastische model simuleert de dynamica van MRSA in 3 ziekenhuizen, die zowel intensive care (IC) als gewone afdelingen bevatten. Heropname van dragers van MRSA is onderdeel van het model en de patiëntenstromen zijn gebaseerd op observationele data. Zonder additionele interventies is de MRSA prevalentie 5% in het ziekenhuis als geheel en 20% in IC’s. De kosten van interventies en infecties komen uit de literatuur. We vergelijken 4 S&I strategieën met de situatie zonder additionele interventies: S&I van patiënten met een geschiedenis van MRSA dragerschap (hoog-risico patiënten); S&I van hoog-risico patiënten en IC opnames; S&I van hoog-risico patiënten en frequent ziekenhuisbezoek “frequente patiënten”; S&I van alle ziekenhuisopnames (universele screening). Het effect van isolatie op besmettelijkheid varieert van 10%, 25%, 50% tot 100%. Het model voorspelt dat S&I van hoog-risico patiënten en S&I van hoog-risico patiënten en IC-opnames de meest kostenbesparende strategieën zijn en ook het snelst de investering terug verdienen. Als het effect van isolatie laag is zullen universele screening en S&I van frequente en hoog-risico patiënten niet kostenbesparend worden. Universele screening voorkomt nauwelijks meer infecties dan S&I van frequente en hoog-risico patiënten, maar de kosten zijn wel hoger. Of een interventie kostenbesparend wordt binnen 10 jaar hangt sterk af van de kosten per infectie op een IC, de kosten van een screening en het effect van isolatie.

**Abstract in Russian**

Во многих странах частота внутрибольничных инфекций, вызванных устойчивыми к антибиотикам бактериями, такими как метициллин-резистентный стафилококк (methicillin-resistant *Staphylococcus aureus; MRSA)*, остается высокой. Скрининг пациентов с последующим введением карантина для выявленных носителей MRSA (S&I) показал себя успешным средстовм контроля во многих, но далеко не во всех условиях. Более того, несмотря на широкое применение различных стратегий контроля, имеется деффицит исследований направленных на сравнение эффективности и стоимости различных стратегий контроля. В данном исследовании, посредством математической модели, оценены эффективность и стоимость различных S&I стратегий и определены параметры, наиболее значимые для определения оптимальной стратегии.

В данной работе использована динамическая стохастическая модель, в которой описаны три больницы с палатами общего типа и палатами отделения реанимации и интенсивной терапии (ОРИТ), а так же учтена возможность повторной госпитализации носителей MRSA. Моделирование потока пациентов между общими палатами и палатами интенсивной терапии основано на реальных данных. Изначальный уровень MRSA был принят равным 20% в ОРИТ и 5% в среднем по больнице; диапазон частоты заболеваний среди носителей и диапазоны расходов основаны на опубликованных данных. В данном исследовании произведено сравнение эффективности и финансовых затрат четырех S&I стратегий: 1) S&I пациентов, ранее выявленых как носителей MRSA, 2) S&I пациентов, ранее выявленых как носителей MRSA и всех пациентов при приеме в ОРИТ, 3) S&I часто госпитализируемых пациентов и пациентов, ранее выявленых как носителей MRSA, 4) S&I всех пациентов при приеме в больницу. Была исследована эффективность принятых мер карантина равная 10%, 25%, 50% и 100%. Посредством нашей модели предсказано, что наиболее оптимальными по соотношению стоимости и эффективности являются две стратегии: S&I пациентов, ранее выявленых как носителей MRSA, а так же S&I всех пациентов при приеме в ОРИТ и пациентов, ранее выявленых как носителей при приеме в палаты общего типа. Моделью также предказана наиболее быстрая окупаемость этих двух стратегий. При низкой эффективности карантина стратегии S&I всех часто госпитализируемых пациентов и S&I всех пациентов при приеме в больницу могут никогда не окупиться. При этом предсказано, что, при большей стоимости, стратегия S&I всех пациентов при приеме в больницу вряд ли предотвратит заметно большее количество развившихся заболеваний, чем S&I часто госпитализируемых пациентов. Окупаемость в течение 10 лет любой S&I стратегии существенно зависит от величины дополнительных затрат на каждое развившееся заболевание в ОРИТ, стоимости скрининга и эффективности мер карантина.
